# Preliminary Investigations into the Use of Amylases and Lactic Acid Bacteria to Obtain Fermented Vegetable Products

**DOI:** 10.3390/foods10071530

**Published:** 2021-07-02

**Authors:** Ina Vasilean, Iuliana Aprodu, Maria Garnai, Valeriu Munteanu, Livia Patrașcu

**Affiliations:** 1 Faculty of Food Science and Engineering, Dunarea de Jos University of Galati, 111 Domneasca Str, 800008 Galati, Romania; ina.vasilean@ugal.ro (I.V.); iuliana.aprodu@ugal.ro (I.A.); maria.garnai@ugal.ro (M.G.); 2 Cross-Border Faculty, Dunarea de Jos University of Galati, 111 Domneasca Str, 800008 Galati, Romania; munteanu.valeriu1994@gmail.com

**Keywords:** enzyme hydrolysis, vegetable milk substitutes, rheological behavior, three-interval thixotropy test, antioxidant activity

## Abstract

Legumes are valuable sources of proteins and other functional components. However, the high starch content can be an impediment in developing new vegan food formulations. Enzyme-assisted hydrolysis was used to hydrolyze the starch from chickpea and broad bean vegetable milk to further develop vegetable lactic acid-fermented products. The antioxidant activity of legumes was tested, and it was observed that the overall antioxidant activity (DPPH radical scavenging ability) significantly increased after enzyme-assisted hydrolysis while total phenols content decreased. The obtained vegetable milk was then fermented using exopolysaccharides-producing lactic acid bacteria. A significant decolorization was observed after fermentation in the case of broad bean-based products. Rheological behavior of the fermented products was determined using small amplitude oscillatory measurements and the three-interval thixotropy test. Results showed higher complex viscosity values for broad bean-based products, which displayed a weak gel-like structure. The starter cultures used for vegetable milk samples fermentation influenced the resistance to flow.

## 1. Introduction

Plant-based milk or vegetable milk substitutes (herein referred to as ‘vegetable milk’) are water-soluble extracts based on vegetables, legumes, cereals, pseudocereals, or nuts [1], and have become very popular among consumers due to the numerous health-related benefits they provide. 

Legumes are valuable sources of functional components such as proteins, carbohydrates with low glycemic load, prebiotic oligosaccharides, dietary fibers, vitamins, minerals, and phenolic compounds [2,3]. Among legumes, soybean is the most processed legume worldwide, mainly due to its specific chemical composition consisting of low starch, high protein, and high lipid contents. In soybean, starch content is less than 1%, with most carbohydrates being cellulose, pectic polysaccharides, other non-cellulosic polymers, and free sugars such as sucrose, stachyose and raffinose [4]. Non-soy legume products are more difficult to process, mostly because of the high starch content, which absorbs a high amount of water, resulting in thickening of the composition. The functionality of the starch varies depending on the source [5]. Generally, cereal starches have an A-type pattern. Some tuber starches such as potato and cereal starches, which are rich in amylose, yield the B-type starch pattern, while legume starches present mostly a C-type pattern [6,7]. Legume starches are characterized by high amylose content of up to 65% [8], presenting a high ordered structure of the crystalline granules [9]. In particular, the *Phaseolus vulgaris* starches present low swelling power and solubility, resistance to α-amylase attack, high gelatinization temperature, and stable amylographic viscosities [9]. Therefore, obtaining vegetable milk and derived products out of legumes other than soybean is a rather difficult task.

One efficient way of enhancing functional properties of legumes, such as to assure their utilization in food industry, is the hydrolysis treatment of starch-containing flours with acids [10] or amylolytic enzymes [11].

Enzymatic hydrolysis can be naturally achieved during the crop sprouting process. Germination has been found to efficiently enhance the functional properties of crops and legume flours [12,13,14]. Germination enhanced the water [14] and fat absorption capacities and the emulsifying capacity of legume flours [12], favoring the antioxidant capacity. However, the process is time-consuming, also requiring a vast infrastructure. Moreover, native and germinated legume seeds were found to contain only β-amylases [15]. Thus, α-amylases breaks down long-chain carbohydrates; yielding maltotriose and maltose from amylose, maltose, and glucose; and “limit dextrin” from amylopectin. Then, β-amylases act on the nonreducing ends of the starch polymer chains [15,16]. Finally, amyloglucosidases, known as glucoamilases, yield glucose and a low quantity of dextrins [15,16].

In vitro or assisted hydrolysis with amylolytic enzymes is a more approachable technique that can assure starch degradation into smaller molecules, allowing legume processing into a more variable range of food products. It can be used as an alternative to other methods for improving the technological functionality of legumes. For instance, to support the bacterial growth for obtaining a yogurt-like product from lupin seeds, Jiménez-Martínez et al. [17] fortified vegetable milk with lactose and sucrose.

The aim of the present study was to determine the impact of α-amylases and amyloglucosidase on the antioxidant activity, physical-chemical, and rheological properties of the fermented product obtained from vegetable milk based on broad bean and chickpea.

## 2. Materials and Methods

Broad bean and chickpea seeds were purchased from a local market in Galati, Romania. The physical and chemical analysis were performed on the legume flour samples prepared by grinding the seeds using a laboratory mill (WZ-2, Sadkiewicz Instruments, Bydgoszcz, Poland).

### 2.1. Proximate Composition of Legume Flours

The proximate composition of the studied legumes was determined as follows. First, the moisture content was determined using the AACC 44–51 method [18], and the protein content was determined through the semimicro-Kjeldahl method (Raypa Trade, R Espinar, SL, Barcelona, Spain). For these compositions, we used the nitrogen conversion factor of 6.00 and determined the fiber content through the AOAC official method 962.09 [19], using the Gerhardt Fibertech equipment (C. Gerhardt GmbH & Co. KG, Königswinter, Germany). The ash content was determined using the SR ISO 2171: 2002 method [20]. The amount of reducing sugars was determined using the 3,5-dinitrosalicylic acid (DNS) assay [21].

### 2.2. Production of the Vegetable Milk

Broad bean and chickpea seeds (200 g each) were washed in running water and sanitized by soaking for 15 min in a 70% aqueous alcohol solution. Clean seeds were further allowed to swell in tap water for 12 h. At the end of the soaking step, any water excess was discarded. The soaked seeds were finely grinded with 750 mL of tap water for 10 min in a TM5 Thermomix blender (Vorwerk Elektrowerke GmbH & Co., Wuppertal, Germany). Vegetable milk was obtained by straining the slurry though a cotton gauze. The dry matter content of the broad bean and chickpea-based milk samples was 9.68 ± 0.05% and 10.37 ± 0.01%, respectively.

### 2.3. Enzymatic Hydrolysis

Obtained vegetable milk samples were subjected to hydrolysis with amylolytic enzymes according to the procedure of Adthalungrong and Temviriyanukul [22] with modifications. First, α-amylase (BAN 240L, Novo Nordisk) was added at a ratio of 1 g/100 g starch. The mixture was heated to 70 °C in the TM5 Thermomix blender (Vorwerk Elektrowerke GmbH & Co., Wuppertal, Germany) with continuous stirring. The reaction was allowed to take place for 1 h. Afterward, the temperature was adjusted to 60 °C, and amyloglucosidase (SAN Super 240L, Novo Nordisk, Copenhagen, Denmark) was added at a level of 1 g/100 g starch. The reaction was allowed to take place for another hour. At the end of the hydrolysis step, the inactivation of enzymes was performed by maintaining a temperature of 90 °C for 10 min. 

### 2.4. Lactic Acid Fermentation

Two different starter cultures (SC) were used for vegetable milk fermentation, in agreement with the specifications of manufacturer, YF-L 812 (Christian Hansen, Hoersholm, Denmark). The start cultures were a yogurt starter culture containing *Streptococcus thermophilus*, *Lactobacillus delbrueckii* subsp. Bulgaricus (SC1), and a mixture of *Lactobacillus casei* and XPL-1 (Christian Hansen, Denmark), which is a mixed culture containing *Lactococcus lactis* subsp. cremoris, *Lactococcus lactis* subsp. lactis, *Leuconostoc* species, *Lactococcus lactis* subsp. lactis biovar. Diacetylactis, and a *Streptococcus thermophilus* strain (SC2), added for texture improvement [23]. The enzyme-treated vegetable milk samples were inoculated with SC1 and SC2 and further incubated at 43 °C for 10 h to reach a pH of 4.6. The fermented samples obtained from broad bean (B1 and B2 fermented with SC1 and SC2, respectively) and chickpea (Ch1 and Ch2 fermented with SC1 and SC2, respectively) were stored at 4 °C prior to analyses.

### 2.5. Antioxidant Activity Determination

Vegetable milk samples and fermented products were centrifuged at 9690× *g* for 10 min. The supernatant was collected for further assaying the 2,2-diphenyl-1-picrylhydrazyl (DPPH) free radical scavenging activity and total phenolic content. 

To emphasize the effect of enzymatic hydrolysis on the antioxidant activity of vegetable milk, a parallel sample was prepared in similar conditions (time-temperature) without added enzymes, further termed ‘control.’ 

#### 2.5.1. DPPH Radical Scavenging Ability

The overall antioxidant activity was determined with the DPPH method. In short, 0.1 mL of supernatant was mixed with 3.9 mL of 6 × 10^−5^ M DPPH solution in methanol. After allowing the compounds to react in the dark for 30 min, the absorbance at a wavelength of 515 nm was recorded to quantify the remaining DPPH. The DPPH radical scavenging activity of studied legumes is expressed as an IC50 value, representing the amount of antioxidant (mg of legume flours or yoghurt like product) necessary to decrease the absorbance of the used DPPH solution by 50% [24].

#### 2.5.2. Total Phenolics Content

The Folin-Ciocalteu method was used to determine the concentration of total phenolic compounds. A volume of 0.2 mL extract was mixed with Folin–Ciocalteu reagent (1.5 mL, previously diluted with water 1:10, *v*/*v*). After 10 min of resting at room temperature, 1.5 mL of 60 g/L sodium carbonate was added. The mixture was allowed to rest for additional 90 min, and then the absorbance was read at 725 nm. Ferulic acid was used as reference, and the total phenolic compounds were quantified and reported as mg ferulic acid equivalents (FAE)/g sample.

### 2.6. Titratable Acidity Determination

The titratable acidity of the fermented samples was determined by titration with NaOH 0.1N against phenolphthalein. The pH values were determined using the InoLab pH7100 pH meter (WTW, Weilheim, Germany).

### 2.7. Syneresis Determination

Syneresis phenomenon is defined as the amount of liquid expelled from the gel structure of the fermented samples. Syneresis was determined as g liquid expelled by 100 g fermented product while being centrifuged at 3000× *g* for 5 min.

### 2.8. Color Determination

CIELAB color parameters (L*, a*, b*) of the vegetable milk and fermented products were determined using the CR410 chroma meter (Konica Minolta, Tokyo, Japan), equipped with C illuminant.

### 2.9. Sensory Analysis

The descriptive sensory analysis was performed after 24 h storage at 4 °C. The fermented samples were evaluated in a sensory laboratory under white light. The sensory attributes taken into account were taste, texture, color, flavor, and overall acceptability. A panel consisting of 10 trained members, familiarized with the sensory descriptors and the attribute intensities, conducted the sensory analysis.

### 2.10. Rheological Measurements

Rheological behavior of the fermented samples was studied using a control-stress rheometer (AR2000ex, TA Instruments, Ltd., New Castle, DE USA), equipped with a Peltier jacket temperature control system. A cup and conical cylinder geometry assembly was used with a bob diameter of 28 mm and 42 mm in length. Rheological tests were carried out at 20 °C. The storage modulus (G′) and loss modulus (G′′) were registered. Oscillatory strain sweep test over an oscillatory strain (γ) range of 1.0–100%, at frequency of 1Hz was performed to identify the linear viscoelastic region (LVR) of the samples (where G′, G′′ values are not influenced by the strain magnitude), together with the flow point (at G′- G′′ intersection). The thixotropy phenomenon was observed by applying a 3-interval thixotropy test (3ITT) in low-amplitude oscillatory conditions, as described by Toker et al. [25]. The first reference interval involved applying a strain value of 0.1% (within LVR) for 5 min. During the high-shear interval, the strain was raised to 100% and maintained for 5 min. Finally, a regeneration interval was allowed for 10 min at 0.1% strain. For each sample, the recovery percentage of the structure was determined according to the following equation:

(1) $$\%Rec=\frac{Gf}{Gi}\times 100,$$

where *Gi* represents the average G′ value from the first interval and *Gf* represents the final G′ of the sample from the third interval.

### 2.11. Statistical Analysis

The statistical analysis of the results was carried out using Statgraphics Centurion XVI.I software. The data were subjected to single-factor ANOVA analysis with a significance level of 95.0%. Fisher’s least significant difference (LSD) test at a 95.0% confidence level was used to determine differences between mean values. All analyses were carried out in duplicate and data were reported as mean values ± standard deviation.

## 3. Results and Discussion

### 3.1. Influence of Amylase Assisted Hydrolysis on the Antioxidant Activity of Vegetable Milk and Fermented Products

Broad beans and chickpeas were used to obtain vegetable milk substitutes. The proximate composition of the broad bean and chickpea flours is presented in Table 1. The amylase-assisted hydrolysis of the vegetable milk samples was carried out to increase the amount of sugars useful for fermentation with lactic acid bacteria. Upon hydrolysis, the amount of reducing sugars increased from 2.85 ± 0.03 to 9.33 ± 0.05 g glucose/100 g dw in broad bean samples and from 1.76 ± 0.04 to 11.14 ± 0.02 g glucose/100 g dw in chickpea samples.

The antioxidant activity of the obtained vegetable milk samples is presented in Table 2. When compared to the corresponding control samples, it can be observed that enzymatic hydrolysis determined a significant increase of DPPH radical scavenging ability (*p* < 0.05) for both legumes considered in the study. Regarding the total phenols content, a significant decrease was observed upon hydrolysis for both broad bean and chickpea milk samples (*p* < 0.05). In this respect, Vuorela et al. [26] also reported a significant reduction of the total phenolic content in rapeseed meal after enzymatic hydrolysis.

The vegetable milk samples were further fermented using two different SCs. The obtained fermented products presented a nice texture, specific to the yogurt. A creamier texture was noticed in the case of the broad bean-based fermented products, whereas the chickpea-based samples were more fluid. Regardless of the SC used for fermentation, no dairy notes were noticed in any of the fermented products. The broad bean-based samples presented a more pleasant lactic flavor, while the chickpea-based samples preserved some beany attributes. Moreover, a sour astringent aftertaste was perceived in the case of the chickpea-based samples. Considering the better flavor and the absence of the beany or astringent taste, better acceptability was decided for the broad bean-based fermented products.

The antioxidant activity of the fermented samples obtained from enzymatically hydrolyzed broad bean and chickpea milk is presented in Table 3. Lactic fermentation determined the increase of the DPPH radical scavenging ability (*p* < 0.05), with the exception of the Ch2 sample (chickpea-based vegetable yogurt fermented with SC2). A similar trend was reported by Apostolidis et al. [27], who compared the performance of *Lactobacillus bulgaricus* and *Lactobacillus acidophilus* to ferment the milk and soymilk. A higher increase of the DPPH radical inhibition was obtained for both milk- (by 23%) and soy milk- (by 20%) based products when the fermentation was carried out with *L. acidophilus* compared to the samples fermented with *L. bulgaricus* (increase of 9% for milk and 6% for soy milk). Total phenols content also increased after fermentation (*p* < 0.05) for all samples. These results are partly due to the bacterial enzymes produced during fermentation, which enable the extraction of different biologically active compounds as the result of the disintegration of the plant cell walls. In addition, the lactic acid bacteria may be involved in releasing new biologically active compounds through hydrolysis or in phytochemicals transformation, such as the depolymerization of the high molecular weight phenolic compounds or the release of the aglycons from the glycosylated isoflavones [28]. 

After 2 weeks of storage, the antioxidant activity of all samples significantly decreased (*p* < 0.05), reaching values lower than those found in the vegetable milk subjected to fermentation. On the other hand, after 2 weeks of storage, the total phenols content of the fermented samples resembled the values obtained for the corresponding vegetable milk samples (*p* > 0.05). When comparing the samples obtained from the same type of vegetable milk, no statistically significant differences were found among samples prepared with different starter cultures, neither immediately after fermentation nor after 2 weeks of storage (*p* > 0.05). A similar decrease of the antioxidant activity and total phenolic content was reported by Kim et al. [29] when investigating tea fermentation. The authors explained this decrease by the oxidative degradation of the flavonol glycosides.

### 3.2. Physicochemical Characteristics of the Fermented Products

To study the effect of starter cultures on quality characteristics of the fermented products, acidity, pH, syneresis, and color were determined. Analyzing the titratable acidity results presented in Table 4, there were no significant differences between samples obtained from the same type of vegetable milk fermented with different starter cultures (*p* > 0.05). However, the fermented products obtained from chickpea presented lower titratable acidity values in comparison with the broad bean ones (*p* < 0.05), even if the pH values were similar for all samples (*p* > 0.05). The higher acidity of broad bean products may be attributed to the higher amounts of buffering compounds present in raw seeds. Among legumes, broad bean was reported to contain the largest amounts of Ca^2+^ (103 mg/100 g), phosphorus (421 mg/100 g), and potassium (1062 mg/100 g) [30], whereas chickpea was reported to contain only 57 mg Ca^2+^, 252 mg phosphorus, and 718 mg potassium per 100 g [31].

CIELAB color parameters of the vegetable milk and fermented products are presented in Table 5. In the case of the chickpea-based samples, the lightness values of the vegetable milk and fermented products were similar (*p* > 0.05), whereas in the case of broad bean, the lightness values significantly increased after fermentation (*p* < 0.05). A similar trend was observed for yellowness (b*). In the case of the broad bean-based fermented samples, the b* value significantly increased (*p* < 0.05), while for the chickpea-based samples, the yellowness presented a slight insignificant increase (*p* > 0.05) after fermentation. Indeed, at visual inspection, a grayish color was observed in the case of the broad bean milk, which whitened after fermentation. This decolorization phenomenon could be the result of some combined reactions. First, it can be the result of lipoxygenase activity. Lipoxygenase involvement in flour bleaching through oxidation of chlorophyll and carotenoid pigments is well documented ([32] and cited literature therein; [33]). Broad bean is known to have 50% of the lipoxygenase activity found in soy, and is listed as a legume with medium lipoxygenase activity [34]. In comparison, chickpea was reported among legumes with low lipoxygenase activity [34]. It is worth noting that soy lipoxygenase type-1 activity was reported to be inhibited by calcium ions, whereas lipoxygenase-2 was reported to be calcium-stimulated [32], and broad bean flour is known to have a high amount of calcium in comparison to other legumes. Also, broad bean has higher type-2 lypoxigenase activity compared to type-1 [35]. 

Another responsible mechanism for the decolorization of the broad bean-based fermented products observed in the present study could involve the amylolytic enzymes activity on color pigments. Depending on the variety, the pigmentation of the Vicia faba seed coats is mainly attributed to chlorophyll, anthocyanins, and tannins [36]. In plants, anthocyanins are generally present as glycosides, while the sugar-free residues are known as anthocyanidins [37]. Saigusa et al. [38] stated that the red pigment of aromatic red rice wine was decolorized by enzymatic digestion with the β-glucosidase fractionated from commercial glucoamylase, releasing glucose from the red pigment in the process. A similar effect was observed by Saigusa et al. [39] in a fermented beverage obtained from purple-fleshed sweet potato, where amylolytic enzymes were used for starch hydrolysis. Another cause for decolorization was anthocyanins loss due to polyphenol oxidase activity ([40], cited by [41]). Su & Silva [41] reported a significant loss in anthocyanins after acetic fermentation in blueberry vinegar. On the other hand, the tyrosinase inhibitory activity of broad bean proteins and peptides can also be mentioned. El-Sayed et al. [42] found monophenolase inhibitory peptides in broad bean pods hydrolysates. Karkouch et al. [43] reported that vicilin and legumin B from Vicia faba seeds hydrolysates had tyrosinase inhibitory activity (up to 81%). Tyrosinase is known to catalyze phenolic compounds oxidation to quinones, being responsible for the enzymatic browning of fruits and vegetables [44].

However, considering that the vegetable milk samples were subjected to thermal treatment, only the residual activity of the thermally stable enzymes originating from broad beans should be taken into account.

### 3.3. Rheological Characteristics of the Fermented Products

Low-amplitude oscillatory strain sweep tests and three-interval thixotropy tests were performed to compare the effect of the two lactic acid bacteria cultures on the rheological characteristics of the obtained legume-based fermented products.

**Figure 1:**
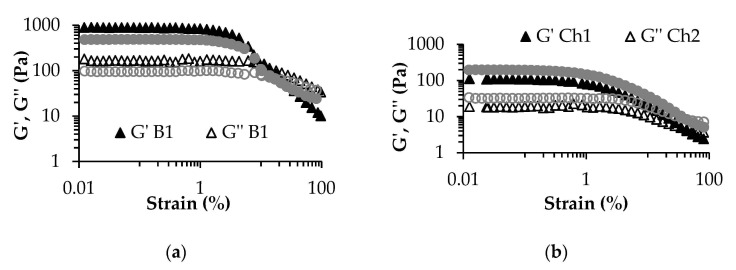
Rheological behavior of (**a**) broad bean- and (**b**) chickpea-based fermented products during low-amplitude oscillatory strain sweep test. B1—broad bean-based product fermented with SC1; B2—broad bean-based product fermented with SC2; Ch1—chickpea-based product fermented with SC1; Ch2—chickpea-based product fermented with SC2.

Figure 1 presents the log-log plot of viscoelastic moduli G′ and G′′ vs. strain%. G′ is associated with the elastic nature of the tested product, whereas G′′ is associated with the viscous nature. A wider LVR (up to approximately 1% strain) was observed in the case of broad bean-based samples (Figure 1a) compared to chickpea-based ones (up to approximately 0.2% strain) (Figure 1b). LVR is the region where the applied strain does not’t affect the internal structure of the tested material. The G′ values in the LVR are usually associated with consistency. In this respect, it could be observed that broad bean-based samples registered significantly higher G′ values in LVR in comparison with chickpea ones (*p* < 0.05). A similar trend was registered for the complex viscosity values in the LVR (Table 6). Higher consistency of broad bean-containing sourdoughs in comparison to chickpea-containing ones was also reported by Patrașcu et al. [14]. No clear correlation between starter cultures and consistency could be observed. Even if G′ and G′′ values appeared slightly higher for B1 in comparison to B2 sample, in the case of chickpea, the behavior was reversed. Either way, the differences (B1vs. B2 and Ch1 vs. Ch2) were statistically insignificant (*p* > 0.05). However, the fermentation with SC2 of both vegetable milk samples showed a better resistance to flow (*p* < 0.05). The G′- G′′ crossover point marks the beginning of flow (flow point) and the destruction of the internal structure of the product. A higher strain value at G′- G′′ crossover is associated with a stronger network. When comparing the two starter cultures used in the study, significantly higher (*p* < 0.05) flow points (strain value at G′- G′′ intersection) were registered for SC2 in the case of both broad bean and chickpea samples (Table 6), while chickpea samples presented a significantly higher resistance to flow (*p* < 0.05). The G′ values of broad bean samples presented a sharp decrease at the end of the LVR, while in the case of chickpea, a smooth transition to viscous behavior was observed (Figure 1). The first case can be associated with a brittle fracturing behavior, while chickpea-based sample presented a creamier structure [45]. The two different structures registered during rheological tests are well correlated with physical appearance of the samples. Upon the visual observation while steering the product with a spoon, broad bean-based fermented products presented a weak but compact gel structure, while chickpea-based products had a dispersion-like appearance. It worth mentioning that, in this respect, Matheis and Whitaker [46] stated that lipoxygenase activity favors the cross-linking of proteins.

**Figure 2:**
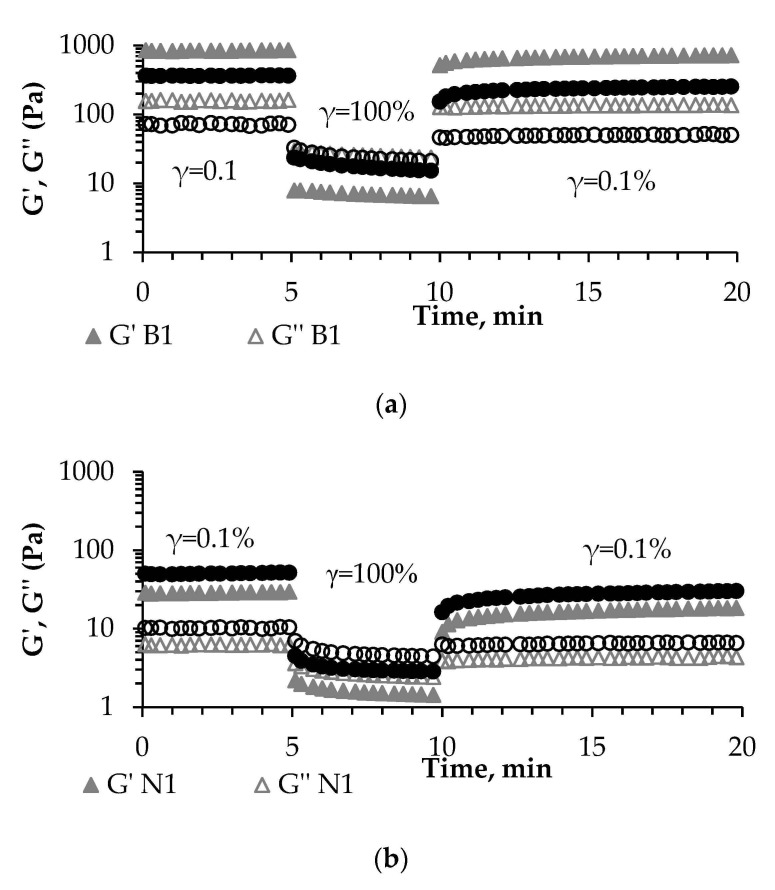
Structure recovering ability of (**a**) broad bean- and (**b**) chickpea-based fermented products determined with 3ITT under low-amplitude oscillatory conditions. B1—broad bean-based product fermented with SC1; B2—broad bean-based product fermented with SC2; Ch1—chickpea-based product fermented with SC1; Ch2—chickpea-based product fermented with SC2.

Regarding the thixotropic behavior and the complete time-dependent recovery of the initial state upon reduction of the load [45], only the B1 sample approached the requirements, with 82% of structure recovery registered during the 10 min of the applied quasistatic state (third region in Figure 2, corresponding to strain value of 0.1%, within LVR).

## 4. Conclusions

The present study aimed to determine some quality characteristics of broad bean- and chickpea-based fermented products. The lactic acid bacteria were used for fermenting the vegetable milk obtained after hydrolysis with amylolytic enzymes. Both enzymatic hydrolysis and fermentation favored the in vitro antioxidant activity of vegetable milk and fermented products. Broad bean-based fermented products registered a higher titratable acidity for similar pH values in comparison to chickpea, as well as a lower syneresis phenomenon. A significant decolorization was observed in the case of the broad bean fermented product, resulting in higher lightness and yellowness values after fermentation. Rheological tests revealed a creamier structure for chickpea-based fermented products and a higher consistency for broad bean-based ones, resembling a weak gel. The SC2 starter culture used for fermentation—a culture with increased exopolysaccharides productivity—resulted in samples with a better resistance to flow, while no correlations could be observed in terms of consistency. Further experiments will deal with the optimization of the enzyme-assisted hydrolysis and fermentation steps while monitoring the functional features and sensory attributes of the fermented products.

## Figures and Tables

**Table 1:** Proximate composition of studied legume flours.

	Water, g/100 g	Ash, g/100 g	Proteins, g/100 g	Fat, g/100 g	Fibers, g/100 g	Carbohydrates *, g/100 g
Broad bean	9.11 ± 0.08	3.68 ± 0.00	24.51 ± 0.80	0.92 ± 0.07	6.61 ± 0.49	55.17
Chickpea	9.53 ± 0.03	2.74 ± 0.00	17.36 ± 0.06	5.6 ± 0.18	2.29 ± 0.31	62.42

* Determined by difference.

**Table 2:** Effect of enzymatic hydrolysis on antioxidant activity of vegetable milk obtained from pulses.

Sample Treatment	IC50 of DPPH, mg D.W.		Total Phenols, mg Ferulic Acid/g D.W.	
	Broad Bean	Chickpea	Broad Bean	Chickpea
Control	4.50 ± 0.06	9.92 ± 0.86	4.59 ± 0.14	2.98 ± 0.13
Hydrolyzed vegetable milk	2.84 ± 0.03	5.99 ± 0.03	3.88 ± 0.04	2.42 ± 0.07

D.W.—dry weight.

**Table 3:** Antioxidant activity of the fermented vegetable products.

Sample	IC50 of DPPH, mg D.W.		Total Phenols, mg Ferulic Acid/g D.W.	
	Initial	2 Weeks of Storage	Initial	2 Weeks of Storage
B1	2.15 ± 0.26 ^a^	3.34 ± 0.02 ^a^	5.68 ± 0.05 ^a^	3.90 ± 0.13 ^b^
B2	2.21 ± 0.08 ^a^	3.82 ± 0.07 ^a^	5.73 ± 0.04 ^a^	3.70 ± 0.08 ^b^
Ch1	5.78 ± 0.10 ^b^	8.27 ± 0.12 ^b^	3.85 ± 0.02 ^b^	2.65 ± 0.10 ^a^
Ch2	5.93 ± 0.02 ^b^	8.86 ± 0.00 ^b^	3.88 ± 0.01 ^b^	2.45 ± 0.02 ^a^

Mean values sharing a lowercase letter within a column are statistically similar at a 95.0% confidence level. D.W.—dry weight, B1—broad bean-based product fermented with SC1; B2—broad bean-based product fermented with SC2; Ch1—chickpea-based product fermented with SC1; Ch2—chickpea-based product fermented with SC2.

**Table 4:** Physicochemical characteristics of fermented vegetable products.

Physicochemical Parameter	Sample			
	B1	B2	Ch1	Ch2
Titratable acidity (ml NaOH 0.1n/100 g)	132.09 ± 1.89 ^a^	124.23 ± 6.71 ^a^	110.23 ± 6.02 ^b^	118.59 ± 1.80 ^b^
pH value	4.24 ± 0.01 ^a^	4.36 ± 0.00 ^a^	4.13 ± 0.02 ^a^	4.00 ± 0.01 ^a^
Syneresis, g liquid/100 g product	35.81 ± 1.15 ^a^	32.14 ± 2.93 ^a^	41.58 ± 1.52 ^b^	41.84 ± 2.34 ^b^

Mean values sharing a letter within a row are statistically similar at a 95.0% confidence level. B1—broad bean-based product fermented with SC1; B2—broad bean-based product fermented with SC2; Ch1—chickpea-based product fermented with SC1; Ch2—chickpea-based product fermented with SC2.

**Table 5:** CIELAB color parameters of obtained vegetable milk and lactic acid fermented vegetable products (yoghurt like).

	Color Parameters		
	L*	a*	b*
Vegetable milk			
Broad bean	57.32 ± 3.57 ^a^	2.01 ± 0.02 ^bc^	4.70 ± 0.36 ^a^
Chickpea	75.87 ± 1.25 ^c^	1.58 ± 0.13 ^a^	17.31 ± 3.43 ^c^
Fermented products—Immediately after fermentation			
B1	71.45 ± 0.14 ^b^	2.32 ± 0.03 ^c^	10.51 ± 0.03 ^b^
B2	71.00 ± 0.49 ^b^	2.12 ± 0.04 ^bc^	10.18 ± 0.03 ^b^
Ch1	76.96 ± 0.51 ^c^	1.44 ± 0.11 ^a^	19.44 ± 0.13 ^c^
Ch2	76.53 ± 0.54 ^c^	1.51 ± 0.35 ^a^	19.19 ± 0.33 ^c^
Fermented products—After 2 weeks of storage			
B1	71.64 ± 1.24 ^b^	2.27 ± 0.40 ^c^	10.6 ± 0.30 ^b^
B2	71.18 ± 0.16 ^b^	2.12 ± 0.65 ^bc^	10.49 ± 0.45 ^b^
Ch1	76.81 ± 0.17 ^c^	1.73 ± 0.79 ^ab^	18.74 ± 1.07 ^c^
Ch2	75.93 ± 1.60 ^c^	1.44 ± 0.28 ^a^	18.71 ± 0.89 ^c^

Mean values sharing a letter within a column are statistically similar at a 95.0% confidence level. B1—broad bean-based product fermented with SC1; B2—broad bean-based product fermented with SC2; Ch1—chickpea-based product fermented with SC1; Ch2—chickpea-based product fermented with SC2.

**Table 6:** Rheological parameters registered during low-amplitude oscillatory strain sweep and 3-interval thixotropy tests for broad bean and chickpea fermented products.

Rheological Parameter	Sample			
	B1	B2	Ch1	Ch2
Complex viscosity in LVR (Pa.s)	112.10 ± 28.82 ^a^	75.11 ± 2.74 ^a^	16.98 ± 3.46 ^b^	31.24 ± 1.89 ^b^
Flow point %γ at G′-G′′ crossover	13.31 ± 0.25 ^a^	16.27 ± 0.09 ^b^	37.55 ± 0.18 ^c^	42.10 ± 0.19 ^d^
% of structure recovery in 3ITT	82.57 ± 2.82 ^a^	62.65 ± 1.19 ^b^	57.85 ± 1.61 ^b^	54.12 ± 2.45 ^b^

Mean values sharing a letter within a row are statistically similar at a 95.0% confidence level. B1—broad bean-based product fermented with SC1; B2—broad bean-based product fermented with SC2; Ch1—chickpea-based product fermented with SC1; Ch2—chickpea-based product fermented with SC2.
